# A Comparative Study of Interdigitated Electrode and Quartz Crystal Microbalance Transduction Techniques for Metal–Organic Framework-Based Acetone Sensors

**DOI:** 10.3390/s18113898

**Published:** 2018-11-12

**Authors:** Karumbaiah N. Chappanda, Mohamed R. Tchalala, Osama Shekhah, Sandeep G. Surya, Mohamed Eddaoudi, Khaled N. Salama

**Affiliations:** 1 Sensors Lab, Electrical Engineering Program, Computer, Electrical and Mathematical Sciences and Engineering Division, King Abdullah University of Science and Technology (KAUST), Thuwal 23955-6900, Saudi Arabia; sandeep.surya@kaust.edu.sa (S.G.S.); khaled.salama@kaust.edu.sa (K.N.S.); 2 Department of Electrical and Electronics Engineering, Birla Institute of Technology and Science, Hyderabad 500078, India; 3 Functional Materials Design, Discovery and Development research group (FMD3), Advanced Membranes & Porous Materials Center (AMPMC), Physical Sciences and Engineering Division, King Abdullah University of Science and Technology (KAUST), Thuwal 23955-6900, Saudi Arabia; mohamed.tchalala@kaust.edu.sa (M.R.T.); osama.Shekhah@kaust.edu.sa (O.S.); mohamed.eddaoudi@kaust.edu.sa (M.E.)

**Keywords:** metal–organic frameworks, IDE capacitors, QCM, comparison, acetone, sensors

## Abstract

We present a comparative study of two types of sensor with different transduction techniques but coated with the same sensing material to determine the effect of the transduction mechanism on the sensing performance of sensing a target analyte. For this purpose, interdigitated electrode (IDE)-based capacitors and quartz crystal microbalance (QCM)-based resonators were coated with a zeolitic–imidazolate framework (ZIF-8) metal–organic framework thin films as the sensing material and applied to the sensing of the volatile organic compound acetone. Cyclic immersion in methanolic precursor solutions technique was used for depositing the ZIF-8 thin films. The sensors were exposed to various acetone concentrations ranging from 5.3 to 26.5 vol % in N_2_ and characterized/compared for their sensitivity, hysteresis, long-term and short-term stability, selectivity, detection limit, and effect of temperature. Furthermore, the IDE substrates were used for resistive transduction and compared using capacitive transduction.

## 1. Introduction

Sensors based on various transduction mechanisms, such as resistive- [1,2,3,4,5,6,7,8,9,10,11,12,13,14], capacitive- [15,16,17,18,19,20,21,22,23,24,25], optical- [26,27,28,29], magnetic- [30], thermal- [31], microwave- [32,33], and resonance-based [34,35,36,37,38,39] techniques, have been applied for detecting a target analyte. Among these sensors, interdigitated electrode (IDE) capacitors and quartz crystal microbalances (QCMs) are the most used because of their small size, low cost, and high sensitivity. Robinson et al. reported a comparison of capacitive and resistive transduction-based sensors using single-walled carbon nanotubes [18]. It was demonstrated that the capacitive detection technique was better (with respect to the resistive technique) in terms of sensitivity, signal-to-noise ratio, hysteresis, and detection range. Recently, Zeinali et al. reported a comparison of IDE and parallel plate capacitor sensors for the detection of polar and nonpolar volatile organic compounds (VOCs) [40]. It was shown that the parallel plate capacitor has higher sensitivity and a better limit of detection. On the contrary, the IDE-based capacitor is better in terms of reproducibility and recovery time. However, scant research has been performed on a detailed comparison of IDE capacitive and QCM (resonator) transduction technique-based sensors. In principle, both transducers consist of a capacitive component. IDE-based capacitive sensors rely on the change in relative permittivity (resulting in a change in capacitance), whereas QCM-based sensors depend on the change in mass (resulting in a shift in resonance frequency). Furthermore, an IDE or QCM substrate alone is unsuitable for sensing applications and requires a material that is sensitive to the target analyte to induce a change in relative permittivity or mass [25,34].

Recently, metal–organic frameworks (MOFs) have garnered great attention because of their high porosity and tunable properties, providing materials with low density, high surface areas, and a large number of active sites for sorption of the analyte of interest [41,42,43,44]. Exceptionally, the chemistry of MOFs offers a limitless number of tunable structures with various surface areas, functionalities, and pore sizes and shapes [45,46]. Recently, many MOFs have shown high affinity in terms of capacity and selectivity for gases, which makes them potential candidates for gas-sensing applications [21,22,25,29,34]. To integrate MOFs for sensing applications, they must be fabricated/grown as thin films [47,48]. Recently, great advances have been made in developing different methods for growing MOFs as thin films on various functionalized supports, such as solvothermal and layer-by-layer methods [21,49,50,51].

In this study, we chose the fabrication of a zeolitic–imidazolate framework (ZIF-8), a subclass of MOFs that resemble zeolites in their topologies, as a sensing layer [52]. Zeolitic–imidazolate framework MOFs have been gathering great attention for various applications because of their chemical and morphological properties. Lu et al. demonstrated a Fabry–Pérot-device-based ethanol and propane sensor using ZIF-8 films grown on a silicon wafer [53]. Furthermore, a ZIF-8 MOF coated on Cu nanoparticles was demonstrated to be sensitive to glucose [54]. Similarly, ZIF-8 is suitable for sensing various analytes such as H_2_O_2_ [55], ascorbic acid [55], H_2_ [56], VOCs [29], and formaldehyde [57,58]. Furthermore, ZIF-8 was used as a template for the synthesis of porous gas sensing material [59]. In addition, ZIF-8 has been shown to be suitable for other applications, such as pervaporation dehydration of alcohols [60], propylene/propane separation [61], hydrogen separation [62], catalyst for Knoevenagel reaction [63], and natural/bio-gas upgradation [64]. Metal oxide coated with ZIF-8 was shown to be sensitive to acetone; however, this was at an elevated temperature [2]. Hromadka et al. demonstrated a highly sensitive VOC sensor (including acetone) using ZIF-8 as the sensing material and long-period grating optical fiber for transduction [29]. However, the transduction mechanism is expensive. Here, it was grown as thin films on two types of electrodes, namely, IDE capacitors and QCMs, which were then used for a comparative study by applying them to sensing a VOC, namely, acetone. 

## 2. Materials and Methods

### 2.1. Material Preparation and Characterization

**Figure 1:**
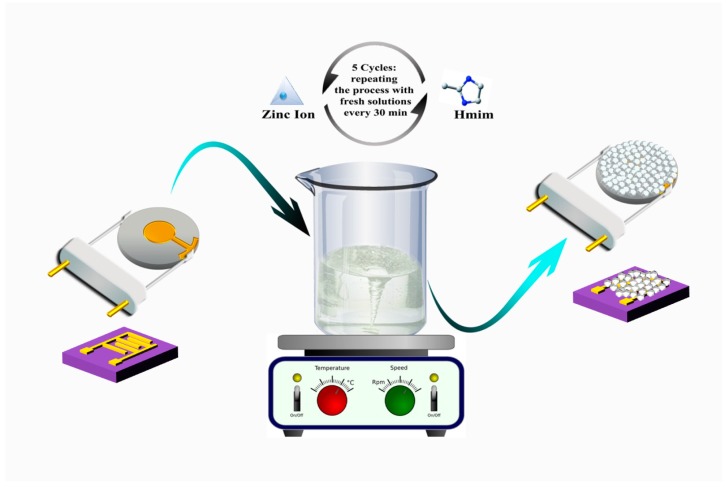
Schematic showing the mechanism used for growing zeolitic–imidazolate framework (ZIF-8) thin films on sensing substrates. The films were deposited by cyclic immersion in a 10-mL mixture of 25 mMol zinc nitrate hexahydrate (5 mL) and 50 mMol 2-Methylimidazole methanolic (5 mL) precursor solutions of [65].

The first type of transducer (IDE capacitors) was fabricated using a 4-in p-type low-doped silicon wafer (>100 Ω·cm) as the substrate. The wafer was degreased by being ultrasonicated in acetone, ethanol, and deionized water, followed by blow-drying in N_2_ gas. Subsequently, the wafer was subjected to wet thermal oxidation at 1100 °C for 60 min to grow 1-µm-thick oxide. Next, 30/250 nm of Ti/Au thin film was sputtered, deposited, and patterned using photolithography and argon ion sputter etching processes. The wafer was then diced into 5-mm dies. The fingers of the IDE were designed with a gap of 5 µm and width of 4 µm. Two IDE substrates (named Cap-1 and Cap-2) were used in this study. For the second transducer, 10 MHz commercial QCMs from Open QCM were used. Two QCM substrates (named QCM-1 and QCM-2) were used for the study here. The thickness of the quartz was approximately 147 µm with a device sensitive area measuring 6 mm in diameter. Two samples of each type of sensor were used to check for reproducibility of the sensor fabrication. Both the diced IDE chips and QCMs were degreased by being ultrasonicated in acetone, ethanol, and deionized water. Cyclic immersion in methanolic precursor solutions technique was used to grow the ZIF-8 thin films onto the devices. Zinc nitrate hexahydrate (25 mMol, Zn(NO_3_)_2_·6H_2_O, 99%) and 2-Methylimidazole (50 mMol, Hmim, 99%) were separately dissolved in 100 ml of methanol solvent. The two QCMs and two IDE capacitors were immersed in a fresh mixture of 1:1 solution (5 mL each) of the metal source and imidazolate linker at room temperature for 30 min with continuous stirring, through which the mixture turned turbid. The transducers were then washed in methanol and dried under N_2_ flow. Thin films were obtained by repeating the process for five cycles with the use of fresh mixture solutions in each cycle [65]. The schematic of the ZIF-8 thin film growth process is shown in Figure 1. The deposited thin films were characterized for their crystalline properties using a PANalytical X’Pert PRO MPD X-ray diffractometer (XRD) at 45 kV, 40 mA for Cu Kα (λ = 1.5418 Å) from 5° to 35°. The thickness and morphology of the deposited films were measured using an FEI Helios NanoLab 400S scanning electron microscope (SEM).

### 2.2. Experimental Setup

**Figure 2:**
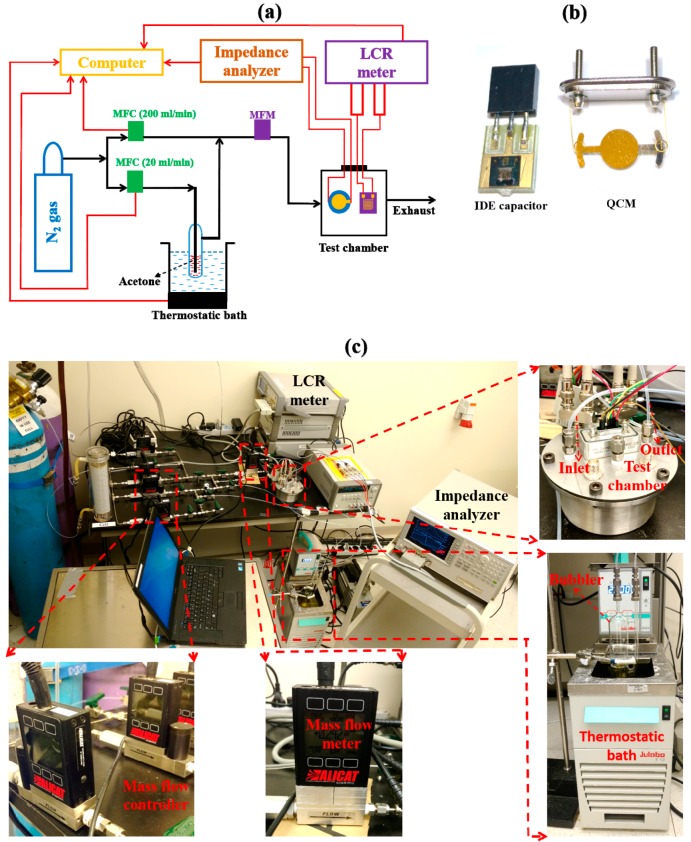
Experimental setup for gas sensing. (**a**) Schematic of the experimental setup used for characterizing and comparing the gas sensors. (**b**) Digital image of the capacitive and quartz crystal microbalance (QCM) substrates coated with ZIF-8 thins film. (**c**) Digital image of the experimental setup.

The fabricated sensors were characterized in a LabVIEW automated custom-built sensor characterization setup at atmospheric pressure [16,66,67,68]. The schematic of the gas sensing setup is shown in Figure 2a, which consisted of two mass flow controllers (MFCs) from Alicat Scientific Inc. (Tucson, AZ, USA) for passing acetone vapor with N_2_ carrier gas (max flow of 20 cm^3^/min) and pure N_2_ gas (max flow of 200 cm^3^/min). Flexible perfluoroalkoxy alkane tubing and stainless-steel pipes with plugs from Swagelok were used as delivery lines. A thermostatic bath (Chiller F12-MA from Julabo GmbH, Seelbach, Germany) set at 22 °C with a glass bubbler was used for generating acetone vapor (by bubbling N_2_ gas). The sensors were tested in a custom-built air-tight chamber with a volume of approximately 400 cm^3^. The temperature in the test chamber was approximately 22 ± 0.5 °C all of the time unless specified. The devices were probed through a XAVAC 15 M hermetic feedthrough connector from Positronic (Springfield, MO, USA). The change in capacitance/resistance of the IDE substrates was monitored using an E4908A LCR meter from Keysight Technologies (Santa Rosa, CA, USA). Unless specified, 200 mV_RMS_ and 100 kHz of alternating current (AC) were used to actuate the IDE substrate. The data points were collected every second using the LabVIEW interface. The shift in resonance frequency of the QCMs was monitored using an E4990A impedance analyzer from Keysight Technologies (Santa Rosa, CA, USA). AC frequency sweeps in the forward direction at 200 mV_RMS_ were used to actuate the QCMs. The data points were collected at the end of every sweep. A digital image of the IDE-capacitor- and QCM-based sensors coated with ZIF-8 thin films is shown in Figure 2b. In case of measurements at elevated temperatures, a hot plate from Torrey Pines Scientific (EcoTherm HS60, Solana Beach, CA, USA) was used for heating from underneath the chamber. A commercial temperature sensor (LM35DZ/NOPB) from Texas Instruments (Dallas, TX, USA) was used to monitor the temperature inside the test chamber. To minimize the errors caused by the temperature gradient inside the chamber, the devices under testing and the commercial temperature sensor were placed at the same distance from the hot plate. A commercial humidity sensor (HIH-4000-003) from Honeywell (Morris Plains, NJ, USA) was used as a reference during humidity measurements. A digital image of the gas sensor characterization setup is shown in Figure 2c. All the measurements were done at least three times except for the long-term and short-term stability tests. Please note that additional components are shown in Figure 2c, which were not used for characterizing the sensors reported herein.

## 3. Results and Discussions

### 3.1. Comparison of IDE-Capacitive- and QCM-Based Sensing

The change in mass on the surface of a QCM is related to the shift in resonance frequency using the Sauerbrey equation [69], which is written as follows:

(1) $$\Delta m=-(\frac{a\sqrt{\rho_{q}\mu_{q}}}{2f_{0}^{2}})\Delta f$$

where Δ*m* is the change in mass on the surface of the QCM, a is the surface area of the gold-coated region of QCM, *ρ_q_* is the density of quartz, *µ_q_* is the shear modulus of quartz, *f*_0_ is the resonance frequency of blank QCM, and Δ*f* is the shift in resonance frequency caused by change in mass. In case of IDE capacitors, the capacitance reactance at drive frequency *f* using closed-form solution with *N* number of electrodes (*N* > 3) [70,71] is calculated as follows:

(2) $$X_{C}=\frac{1}{2\pi fC_{IDE}}$$

where *C_IDE_* is calculated using

(3) $$C_{IDE}=(N-3)\frac{C_{I}}{2}+2\frac{C_{I}C_{E}}{C_{I}+C_{E}}$$

where *C_I_* and *C_E_* are calculated using

(4) $$C_{I}=\varepsilon_{0}L(\frac{K(k_{I\infty })}{K(k_{I\infty }^{\prime })}+(\varepsilon_{1}-1)\frac{K(k_{I,1})}{K(k^{\prime }{}_{I,1})}+\varepsilon_{S}\frac{K(k_{I\infty })}{K(k^{\prime }{}_{I\infty })}$$

(5) $$C_{E}=\varepsilon_{0}L(\frac{K(k_{E\infty })}{K(k_{E\infty }^{\prime })}+(\varepsilon_{1}-1)\frac{K(k_{E,1})}{K(k^{\prime }{}_{E,1})}+\varepsilon_{S}\frac{K(k_{E\infty })}{K(k^{\prime }{}_{E\infty })}$$

where *L* is the length of electrodes, *ε*_1_ is the relative permittivity of the layer coated onto the IDE, *ε_S_* is the relative permittivity of the substrate, *ε*_0_ is the relative permittivity of free space, *K*(*k*) is the complete integral of the first kind with modulus *k*, $k^{\prime }=\sqrt{1-k^{2}}$, $k_{I\infty }=\sin(\frac{\pi n}{2})$, $k_{E\infty }=\frac{2\sqrt{n}}{1+n}$, $n=\frac{2W}{\lambda }$, λ=2(W+G), *W* is the width of each electrode, and *G* is the gap between the IDE.

**Figure 3:**
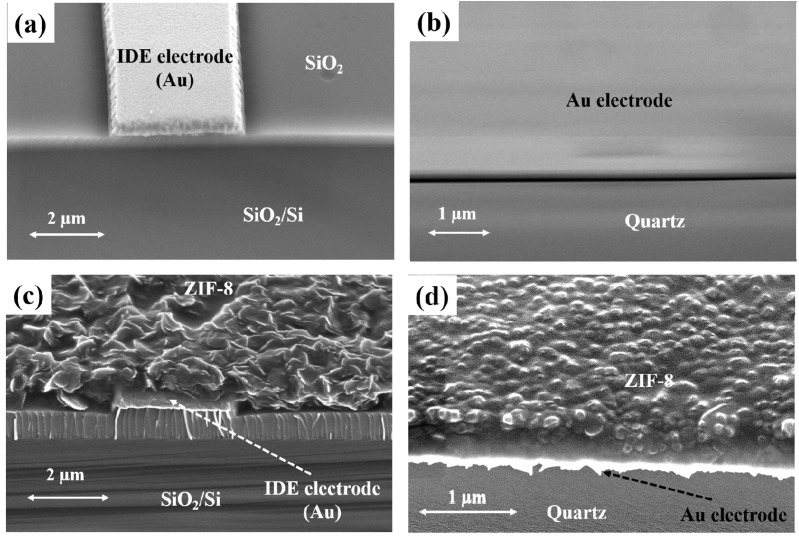
SEM micrographs before and after coating of the ZIF-8 film onto the sensing substrates obtained at a 45° tilt angle. (**a**) Uncoated interdigitated electrode (IDE) capacitive substrate. (**b**) Blank QCM substrate coated. (**c**) IDE capacitive substrate coated with ZIF-8 film. (**d**) QCM substrate coated with ZIF-8 film.

**Figure 4:**
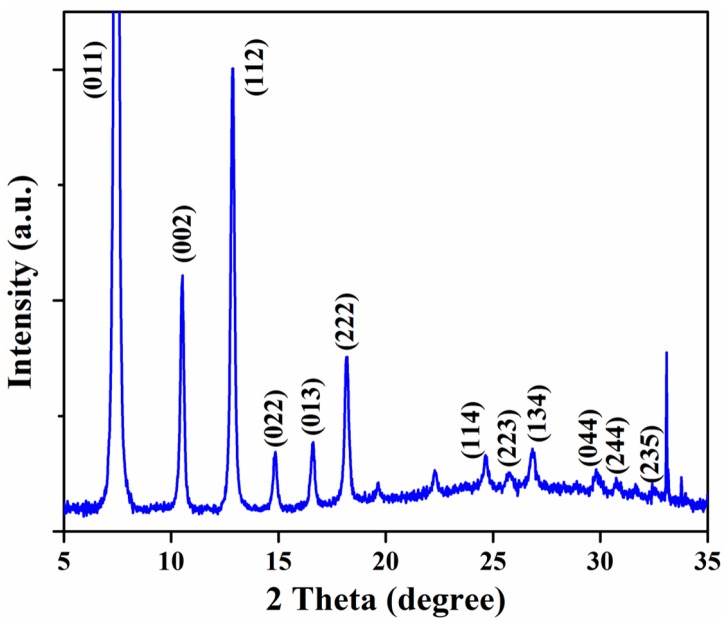
Shows the XRD pattern of a ZIF-8 thin film grown using the cycle method.

The SEM micrographs of uncoated IDE and QCM substrates are shown in Figure 3a,b, respectively. The morphology of the ZIF-8 thin films coated onto the capacitor and QCM characterized using SEM is as depicted in Figure 3c,d, respectively. The average film thicknesses were found to vary between 0.9 and 1.1 μm, and the resulting films on the QCMs were slightly thinner than on the IDE substrates. This may be because of the presence of a higher surface area/roughness provided by the IDE substrate. A higher surface area provides additional nucleation sites for MOF growth compared with the planar/smoother surface of QCM substrates. Furthermore, compared with the QCM substrates, the IDE substrates resulted in ZIF-8 MOF with relatively larger grains of varying size. The thickness and mass of ZIF-8 films deposited onto the device sensitive area of the characterized devices are provided in Table 1. The mass of ZIF-8 MOF on QCM substrates was estimated by measuring the change in the resonance frequency before and after depositing the films. The density of these films was approximately 1.2 g/cm^3^. The mass of ZIF-8 MOF deposited on the IDE substrates was calculated based on the average film density estimated from the QCM substrates. Ideally, sensing substrates with same thickness are better suited for comparative studies but were limited due to our thin film growth technique. The XRD pattern of ZIF-8 film for two theta values between 5° and 35° is shown in Figure 4. The presence of high intensity 011, 002, 112, 022, 013, and 222 peaks verified the formation of ZIF-8 [48,50]. The peaks were well defined, indicating the presence of highly crystalline ZIF-8 thin films. 

**Figure 5:**
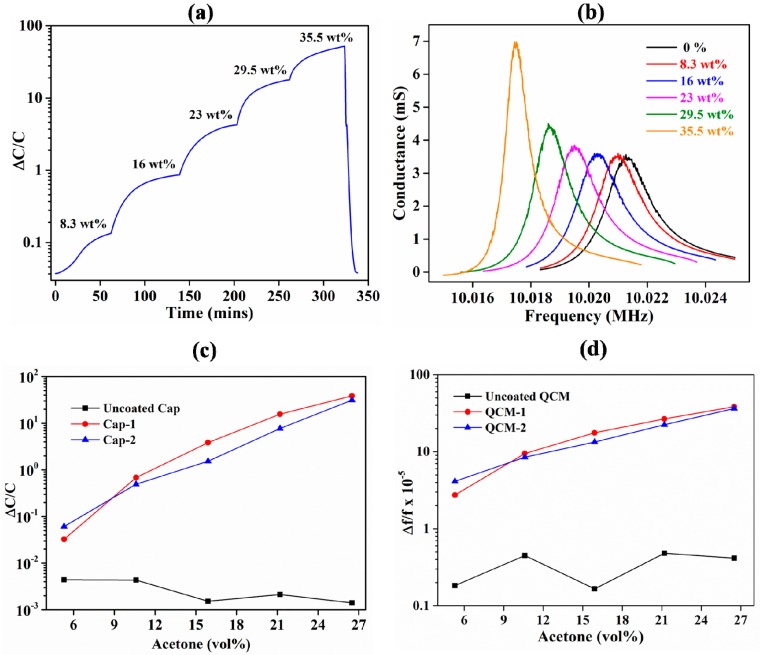
Response of the ZIF-8-coated and uncoated sensors to acetone gas. (**a**) This shows the typical response of the ZIF-8-coated capacitive sensors. (**b**) This shows the typical response of the ZIF-8-coated QCM-based sensors. (**c**) This shows the sensitivity of the two ZIF-8-coated (Cap-1 and Cap-2) and uncoated IDE capacitive sensors. (**d**) This shows the sensitivity of the ZIF-8 coated (QCM-1 and QCM-2) and uncoated QCM-based sensors.

The sensors were exposed to various acetone concentrations ranging from 5.3 to 26.5 vol % in N_2_. A concentration of 5.3 vol % was achieved by setting the flow rates of N_2_ bubbled through acetone and pure N_2_ at 1:5 into the test chamber; 26.5 vol % was obtained by passing N_2_ that was only bubbled through acetone into the test chamber. The typical response and normalized sensitivity of both IDE-capacitor- and QCM-based sensors are shown in Figure 5. The capacitor and QCM had an average sensitivity of 1.3 and 1.4 × 10^−5^ per vol %, respectively. The IDE capacitors showed higher sensitivity compared with the QCMs. This was due to the larger effect of acetone on the dielectric properties (the relative permittivity of acetone is approximately 21) of the capacitor compared with the change in mass when acetone was adsorbed [72]. Therefore, the sensitivity is highly dependent on the transduction mechanism. In addition, various reports have demonstrated that sensitivity is dependent on the amount of sensing material [73,74,75]. Although the thickness of the ZIF-8 films on the QCMs was slightly lower than that on the IDE capacitors, the total mass deposited on the IDE substrates was nearly six times lower than that on the QCM substrates. This shows that IDE capacitive substrates are promising for developing highly sensitive and low-cost sensors. However, both of the transduction techniques showed nonlinear responses. This is because the type of response is mostly dependent on the characteristics of the acetone-sensitive material compared with the sensor’s transduction method or substrate. From the QCM response (Figure 5b), it can be seen that the quality factor of the resonance curve increased with increases in adsorbed acetone. This may be due to the increase in the density of the ZIF-8 films caused by adsorption of acetone into the pores of the MOF, thereby decreasing the damping effect during actuation of the QCM substrate. This is in contrast to previously reported QCM-based gas sensors, wherein the quality factor of the resonance curve reduces with adsorbed gas [34,76]. The mass of acetone adsorbed by the ZIF-8 films was estimated based on the shift in the resonance frequency of the QCM. The mass of the acetone absorbed into the MOF was approximately 0.51, 1.06, 1.67, 2.81, and 4.56 µg when exposed to 5.3, 10.6, 15.9, 21.2, and 26.5 vol % of acetone vapor concertation in N_2_, respectively. It could be observed that the change in absorbed mass with acetone concentration also had nonlinear characteristics, which was similar to its capacitive response. This corroborates that the behavior of the sensor response is mostly dependent on the properties of the sensing material. Based on the estimated mass of adsorbed acetone from the QCM substrates, the mass of adsorbed acetone on the IDE substrates was estimated to be 96, 201, 317, 533, and 866 ng at 5.3, 10.6, 15.9, 21.2, and 26.5 vol % of acetone vapor concertation in N_2_, respectively. Furthermore, the change in relative permittivity caused by acetone adsorbed into ZIF-8 films on the IDE capacitor was estimated using the IDE capacitor model. The change in relative permittivity was 1, 1.5, 2.5, 8.5, and 31.4 at 5.3, 10.6, 15.9, 21.2, and 26.5 vol % of acetone vapor concertation in N_2_, respectively. It could be observed that the change in relative permittivity was nonlinear with linear changes in acetone concentration. Moreover, a previous report showed that the change in trend of relative permittivity is independent of the design of the IDE substrates, further corroborating that the nonlinear behavior is mostly dependent on the sensing material [77]. In addition, the approximate response times of both sensors were less than 60 min. There was a negligible difference in response times because it depends on the sensing material and is independent of the transducing substrates. Notably, the response time reported here does not reflect the true response time. This is because of the flow rate limitation of the vapor delivery system (20 SSCM), which creates the need for a longer time to purge the test chamber (volume 400 cm^3^) with the desired concentration of the acetone. The sensitivity of the two IDE-based sensors (Cap-1 and Cap-2) is shown in Figure 5c. The IDE-based sensors showed slight difference in term sensitivity due to the difference in the thickness of the deposited ZIF-8 film. The sensitivity of the two QCM-based sensors (QCM-1 and QCM-2) is shown in Figure 5d. Similar to the two IDE-based sensors, the QCM-based sensors showed slight difference in term sensitivity due to the difference in the thickness of the deposited ZIF-8 film. The uncoated IDE- and QCM-based substrates showed negligible response to acetone vapors, corroborating the need for ZIF-8 films for sensing acetone.

**Figure 6:**
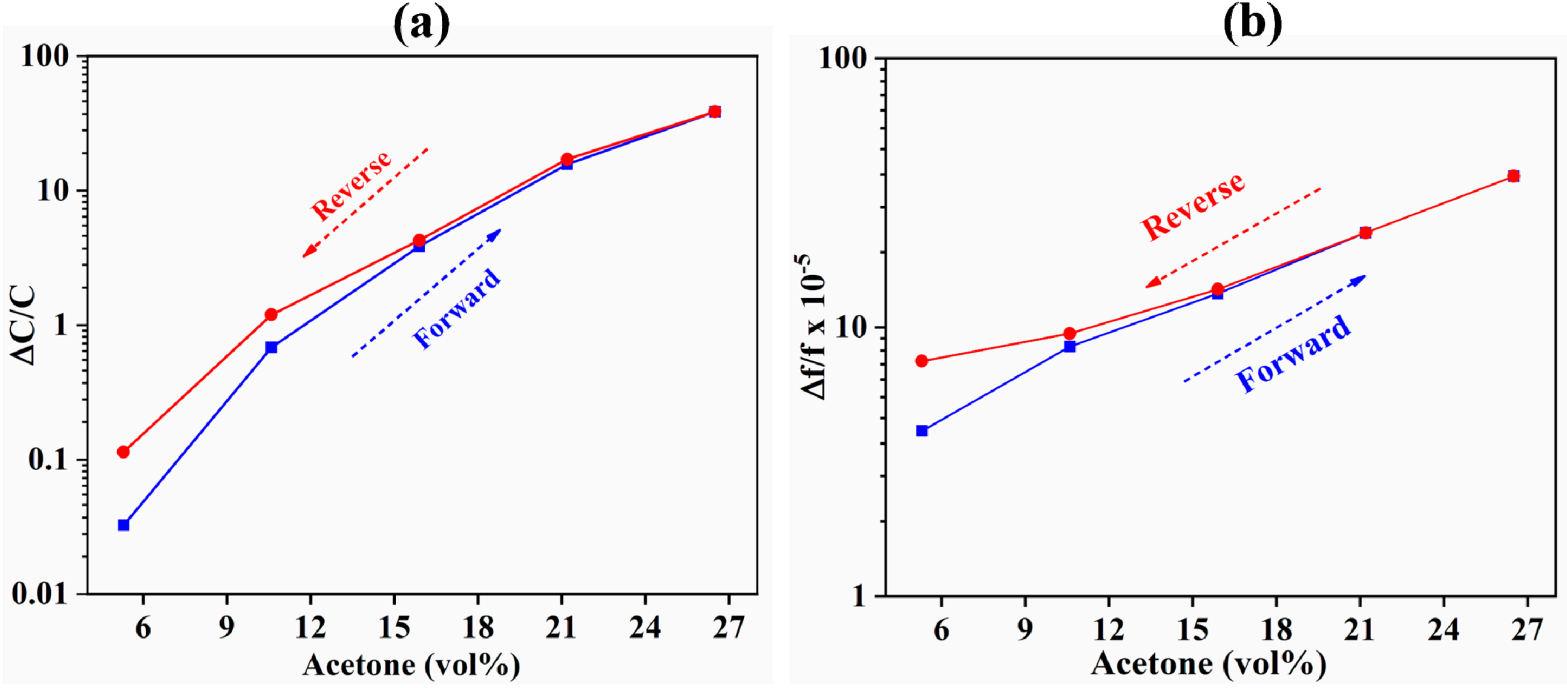
Hysteresis of (**a**) capacitive-based and (**b**) QCM-based sensors. The desorption cycle lagged behind the adsorption cycle by a maximum of approximately 3 vol % at 5.3 vol % of acetone.

Hysteresis of the IDE-capacitive- and QCM-based sensors is shown in Figure 6. Hysteresis is defined as the difference in the response during the adsorption and desorption cycles of the sensor. The hysteresis was measured by increasing the acetone concentration from 0 to 26.5 vol % and then decreasing it from 26.5 back to 0 vol %. The duration of exposure at each level of acetone concentration was 60 min. Both types of sensor showed similar behavior, wherein the desorption cycle lagged behind the adsorption cycle by a maximum of approximately 3 vol % at 5.3 vol % of acetone. The sensors demonstrated near zero hysteresis at 16 vol % and above. This indicated that the hysteresis was caused by the deposited sensing material and was independent of the transduction technique. The lag in desorption cycle may have been caused by the chemical and porous nature of ZIF-8 MOF. The presence of pores in ZIF-8 MOF acted as acetone trap sites, providing greater hindrance during desorption than during the adsorption process. Notably, the lag in desorption cycle at lower acetone concentrations may also be because of the need for a longer time to completely purge the acetone with N_2_ in the test chamber of the characterization setup. The presence of lag/hysteresis could be reduced by heating the ZIF-8 films. This removes the residual trapped acetone and reactivates the sensing films [78].

**Figure 7:**
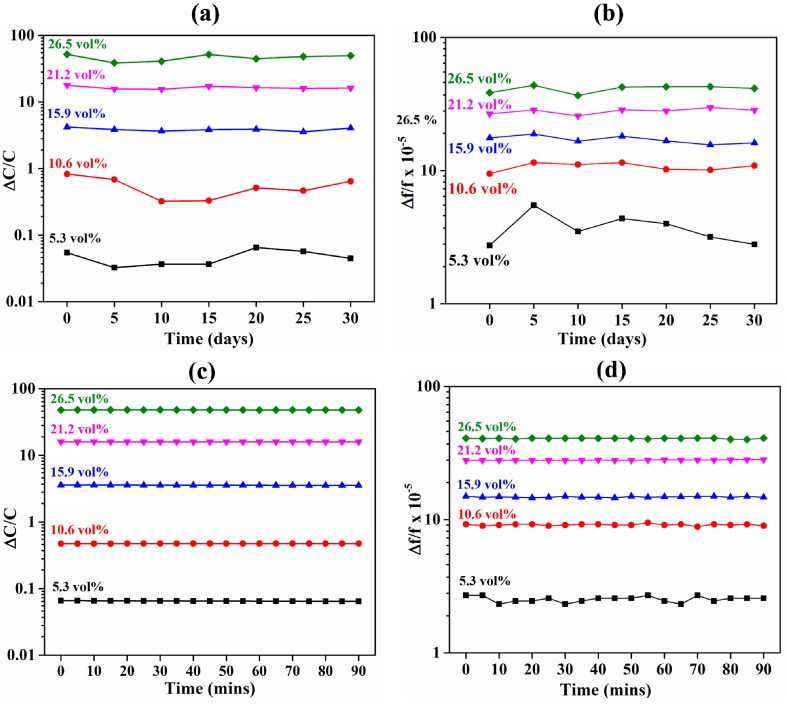
Stability analysis of the ZIF-8-coated sensors to acetone gas. (**a**) Shows the long-term stability of the capacitive sensor for 30 days. (**b**) Shows the long-term stability of the QCM-based sensor for 30 days. (**c**) Shows the short-term stability of the capacitive sensor. (**d**) Shows the short-term stability of the QCM-based sensor.

Next, the sensors were compared for their long- and short-term stability. Long-term stability of the two types of sensor over a period of 30 days with intervals of 5 days is shown in Figure 7a,b. The stability of a sensor depends on the stability of the materials used for constructing it. An ideal reusable sensor should be able to adsorb and desorb the target analyte without changing its chemical properties. Therefore, the long-term stability is dependent on both the sensing material and the transducer. It can be seen that a negligible difference existed between the two sensors in terms of their long-term stability. In this case, the transducing substrates were fabricated using materials (quartz, silicon, gold, and titanium) that are known to be stable under atmospheric conditions and are not affected by acetone. Therefore, the long-term stability was only dependent on the sensing material. It can be seen that ZIF-8 was stable when exposed to acetone and did not show any signs of corrosion or dissolution. The long-term average deviation of the sensors’ output at 26.5 vol % of acetone in N_2_ for over a period of 30 days was 4.4 and 2 × 10^−5^ for IDE- and QCM-based sensors, respectively. Short-term stability of the two types of sensor over a period of 90 min is shown in Figure 7c,d. It can be seen that IDE capacitive sensing had slightly superior short-term stability. Short-term stability is dependent on the sensing material as well as the transduction mechanism. However, the long-term stability of ZIF-8 also implies that the sensing material has a negligible dependence on the short-term stability. However, the transduction mechanism plays a significant role in terms of the short-term stability. The short-term stability of the transducer is dependent on various parameters such as fluctuation in the gas flow rate and pressure. The capacitive transducer is relatively less prone to changes in flow rate and/or pressure, and hence, provides superior short-term stability compared with QCM substrates.

**Figure 8:**
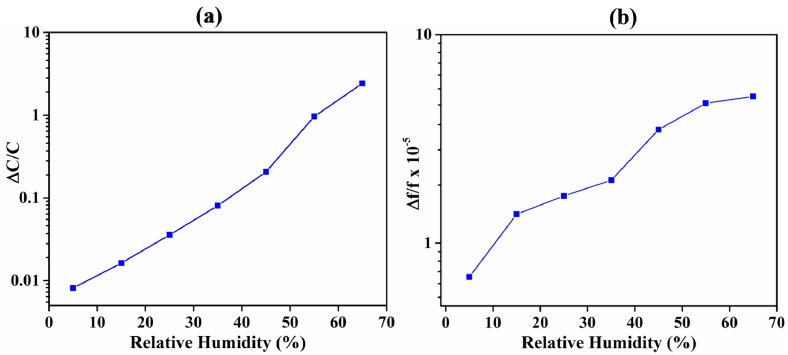
Sensitivity of the ZIF-8-coated sensors to humidity. (**a**) Sensitivity response of the capacitive sensor. (**b**) Sensitivity response of the QCM-based sensor.

A crucial aspect of any gas sensor is its selectivity to the target analyte. The two sensors were compared in terms of their responses to humidity for selectivity. Humidity was chosen because it is most commonly present in air and significantly affects the performance of most gas sensors [21,22]. Notably, although ZIF-8 is shown to be sensitive to other VOCs [29], the purpose here was to study the parameters that affect selectivity and not the selectivity of the material itself. Response of IDE- and QCM-based sensors from 5% to 65% relative humidity (RH) is shown in Figure 8. Both the sensors showed a nonlinear response to a linear change in RH. The IDE and QCM substrates showed a normalized sensitivity of 0.037 and 7.8 × 10^−7^ per % RH, respectively. The responses of the sensors at 65% RH were equivalent to 10.8 and 7.6 vol % of acetone in N_2_ for capacitive and QCM transduction, respectively. At 65% RH, the mass of water adsorbed by the ZIF-8 films on IDE and QCM substrates was 120 and 634 ng, respectively. Although the adsorbed mass of water vapor was greater in QCM-based sensors, it was more selective to acetone compared with IDE transducers with respect to humidity. This is because of the higher relative permittivity (approximately 80) of water vapor [79], which, upon adsorption, causes relatively higher increases in capacitance compared with the same mass of acetone.

**Figure 9:**
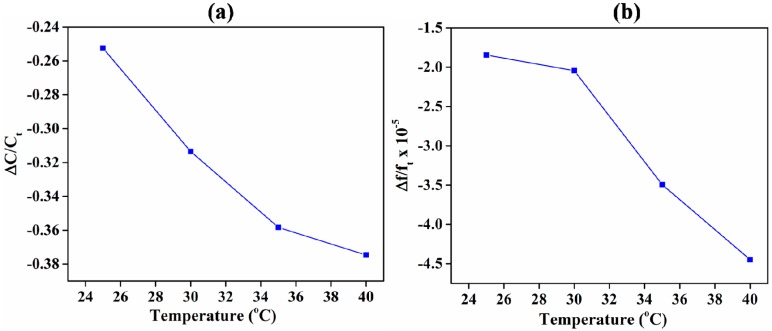
Sensitivity of the ZIF-8-coated sensors to changes in temperature. The acetone was fixed at 15.9 vol %. (**a**) Response of the capacitive sensor. (**b**) Response of the QCM sensor.

One critical environmental factor that affects the performance of a sensor is changes in temperature. The response of the sensors with the rise in temperature from 25 to 40 °C is shown in Figure 9. The concentration of acetone was fixed at 15.9 vol % in N_2_. Both the sensors showed near-linear decreases in sensitivity with increases in temperature. At 40 °C, the change in the device response was equivalent to a reduction in 9 and 6.7 vol % of acetone in N_2_ for IDE- and QCM-based sensors, respectively. This shows that the QCM-based sensor was more immune to changes in temperature than the IDE capacitive sensor. Both the sensing material and the type of transducer are affected with changes in temperature. In the case of the sensing material, increases in temperature weaken the physical bond between ZIF-8 and acetone, thereby resulting in desorption of the analyte. This results in the reduced change in capacitance as well as the resonance frequency of the sensors. In addition, increases in temperature reduce the relative permittivity of acetone [72], thereby further reducing the change in capacitance of IDE-based sensor. In the case of the transduction substrate (based on blank substrate studies), increases in temperature cause the capacitance and resonance frequency to increase. For IDE-based capacitive sensors, increases in temperature result in opposing competitive mechanisms, where capacitance increases because of the substrate and decreases because of the reduced desorption/permittivity of the sensing material. However, the effect of reduced desorption/permittivity is greater than the increase in capacitance of the substrate, resulting in an overall reduced capacitive response of the sensor. In the case of QCM-based sensors, increases in temperature result in complementary effects, where resonance frequency shifts to higher frequencies (a shift to lower frequencies is the required response of the sensor to acetone) due to both the substrate and desorption in the sensing material. Therefore, this results in the reduced sensitivity of the QCM-based sensor with increases in temperature.

**Figure 10:**
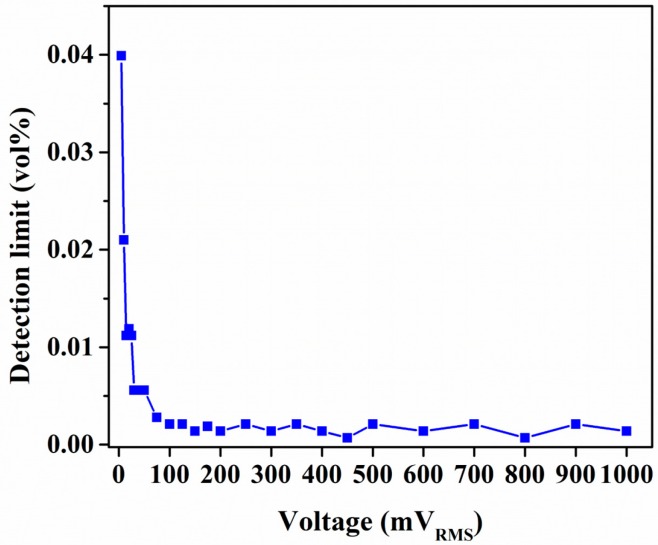
Tunability of the limit of detection by changing the drive voltage in the case of IDE-based capacitive acetone sensor.

One of the crucial parameters of gas sensors is the limit of detection (LOD). The LOD is defined as the ability of a sensor to detect the lowest concentration of the target analyte. The LOD is limited by sensor noise, which is defined by the transducing substrate, sensing material, and experimental setup. The signal-to-noise ratio is estimated using root-mean-square deviation in the baseline response of the sensors [80]. The average signal-to-noise ratio is further multiplied by three, which is considered the minimum detectable output response of the sensor. Here, the LOD was estimated by extrapolating the nonlinear sensitivity response of the sensors (Figure 5c,d) to the minimum detectable output response level. The LOD was approximately 0.0014 and 0.13 vol % for the IDE- and QCM-based sensors, respectively. The lower LOD of the IDE-based capacitive sensor was caused by the superior short-term stability and higher sensitivity of the device. Furthermore, another advantage of IDE-based capacitive sensors is the ability to tune the LOD by changing the actuation voltage. The change in the LOD with increases in actuation AC voltage is shown in Figure 10. It can be seen that at voltages below 100 mV_RMS_, the LOD was significantly affected, whereas it increased with decreases in voltage. At 100 mV_RMS_ and above, there was negligible improvement in the LOD. This is because increases in voltage increased the AC current through the device. A higher current reduces the fluctuation in device response, thereby improving the signal-to-noise ratio and providing an improved LOD. Above a certain threshold current, there is a negligible effect of external noise sources on the sensors’ noise, which results in a negligible change in the LOD. In the case of QCM-based sensors, the increase in actuation voltage has a negligible effect on the LOD because it has a negligible effect on the resonance frequency peaks or quality factor of the resonance curves. 

**Figure 11:**
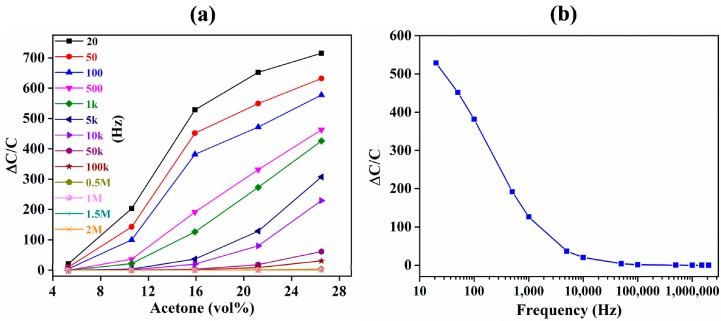
Effect of alternating current (AC) drive frequency on the sensitivity of the ZIF-8-coated IDE-based capacitive acetone sensor. (**a**) Sensitivity response of the sensor to acetone at various AC drive frequencies (20 Hz to 2 MHz). (**b**) AC drive frequency vs. sensitivity of the sensor at 15.9 vol % acetone.

Another added advantage of IDE-based capacitive sensors is the ability to tune their sensitivity in real time by changing the drive frequency. This is limited in QCM-based substrates where the resonance frequency is defined by the thickness of the quartz substrate. Therefore, the sensitivity cannot be tuned in real time because the thickness of the quartz substrate is fixed in each device. The change in the normalized sensitivity with changes in drive frequency from 20 Hz to 2 MHz is shown in Figure 11. The sensitivity decreased exponentially with increases in drive frequency (Figure 11b). At 20 Hz, the average sensitivity was approximately 20 times higher than the sensitivity at 100 kHz. At 100 kHz and above, there were negligible reductions in the sensitivity of the device. The increase in sensitivity is because a lower drive frequency provides a longer time for the polarization of the adsorbed acetone [81]. However, at lower frequencies, the response is relatively noisier because of the larger drift and fluctuation in the output response of the sensor, affecting the LOD as well as the short- and long-term stability of the sensor.

The overall comparison of IDE-capacitive- vs. QCM-based sensors is summarized in Table 2. In summary, IDE-capacitive-based transduction provided the advantages of higher sensitivity, smaller device size, lower LOD, and lower device cost. QCM-based sensors provided other advantages, such as enhanced selectivity with respect to humidity, lower actuation energy costs, and lower temperature-induced shifts in sensitivity. The size of the device was estimated by considering only the area of the acetone-sensitive portion of the sensor. The energy of the device was estimated by modeling the IDE and the QCM as a simple parallel plate capacitor, wherein the energy is given by E = CV^2^/2. For comparison of the device cost, only the substrate cost was considered because only a very small amount of the sensing material was required.

### 3.2. Comparison of Capacitive and Resistive Transduction-Based Sensing Using IDE Substrates

**Figure 12:**
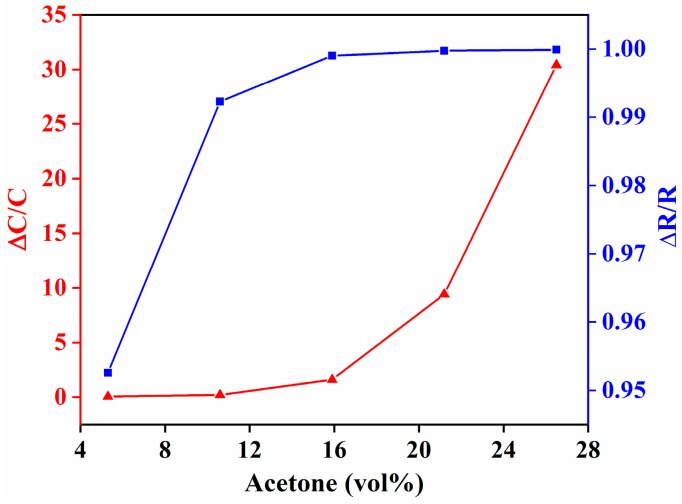
Comparison of changes in resistance vs. capacitance of ZIF-8 film coated onto IDE-substrate-based acetone sensors. The capacitance and resistance values were measured at 100 kHz.

Next, the sensor IDE substrate was used for resistive transduction to sense acetone, and the response was compared with that of capacitive transduction. Comparison of the change in resistance vs. capacitance of the IDE substrate coated with ZIF-8 film is shown in Figure 12. The capacitance showed an exponential increase in the capacitance with linear increases in acetone concentration. In case of resistive transduction, the resistance decreased with increases in acetone concentration. Capacitive transduction showed an increase in sensitivity with increases in concentration of acetone, whereas resistive transduction showed a decrease in sensitivity with increases in concentration of acetone. In other words, the change in sensor response was higher at lower concentrations of acetone for resistive transduction, whereas for capacitive transduction, the change in sensor response was lower at low concentrations of acetone. The average sensitivity for capacitive and resistive transduction was approximately 1.3 and 0.038 per vol % of acetone in N_2_, respectively. This shows that the capacitive transduction was more sensitive to acetone. This is because of the properties of ZIF-8 films; the resistance decreased when exposed to acetone. Therefore, the maximum sensitivity response that can be attained at any concentration of acetone is always less than 1 for resistive transduction, which limits the overall sensitivity of the sensor. 

**Figure 13:**
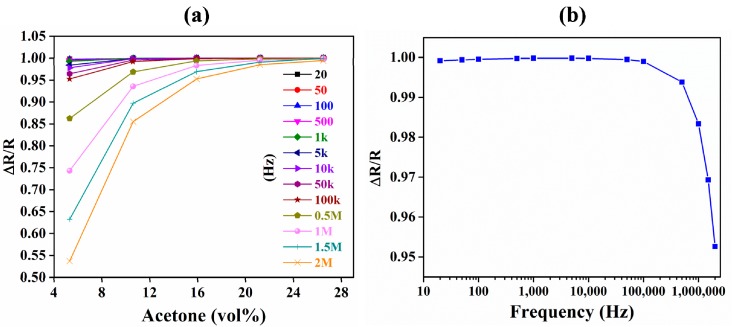
Changes in the device sensitivity vs. actuation frequency for resistive transduction. (**a**) This shows the change in device response at different actuation frequencies. (**b**) This shows the change in device sensitivity with increases in drive frequency at 15.9 vol % of acetone in N_2_.

Similar to capacitive transduction, the sensitivity of resistive transduction can be tuned in real time by changing the drive frequency. The change in sensitivity of resistive transduction with changes in drive frequency is shown in Figure 13. From Figure 13a, it can be seen that the change in the sensor’s response increased with increases in drive frequency. That is, for low concentrations of acetone, the sensor’s change in resistance decreased with increases in actuation frequency. However, irrespective of the actuation frequency, the normalized output response was nearly equal to 1 at 26.5 vol % of acetone in N_2_ (that is, at high concentrations of acetone). This is in contrast to capacitive transduction, where the overall sensitivity decreased with increases in actuation frequency at all acetone concentrations. The changes in device resistive sensitivity with increases in actuation frequency at 15.9 vol % of acetone in N_2_ are shown in Figure 13b. 

### 3.3. ZIF-8 as an Acetone-Sensing Material

Based on the results presented in the abovementioned sections, ZIF-8 is a promising material for developing low-cost and highly sensitive acetone sensors. A comparison of previously reported acetone sensors based on capacitive transduction is shown in Table 3. It can be seen that the device is at least an order magnitude more sensitive than most acetone sensors at room temperature. α-Fe_2_O_3_ was demonstrated to be more sensitive; however, it requires an optimal operating temperature of 250 °C [17]. Although the response time of the ZIF-8 film reported here is low, it was limited due to the vapor generating setup and is expected to be much faster. The comparison of previously reported acetone sensors based on QCM transduction is shown in Table 4; the sensitivity of the device reported here is comparable to most of these sensors. However, the sensitivity can be significantly enhanced by optimizing the ZIF-8 thin film’s thickness and the resonance frequency of the base QCM. Furthermore, Table 5 shows a comparison of previously reported acetone sensors based on resistive transduction; the sensitivity of the device reported here is lower than most of these sensors, showing that it is less suitable for developing highly sensitive sensors. However, most sensors require high operating temperatures [2,5,6,7,8,9,10,11,13] and their sensing performance is limited at room temperature. Moreover, the sensitivity can be improved by optimizing the gap between the fingers of the IDE.

## 4. Conclusions

In this work, we reported a comparative study between IDE and QCM transduction-based acetone sensors using ZIF-8 as the sensing material. The IDE capacitive transduction-based sensors showed the following advantages: higher sensitivity, smaller device size, lower LOD, and lower device cost, whereas QCM transduction-based sensors possessed other advantages, such as superior selectivity with respect to humidity, lower actuation energy cost, and a lower temperature-induced shift in sensitivity. Furthermore, in the case of IDE-based capacitive sensors, the sensitivity and LOD can be tuned in real time by changing the actuation frequency and voltage, respectively. In addition, the IDE substrates were used for resistive transduction and compared using the capacitive sensing of acetone, wherein capacitive transduction was shown to be more sensitive than resistive transduction. Based on previous reports, the capacitance-based sensor has a sensitivity of at least one order of magnitude higher than most sensors. Overall, the ZIF-8 MOF is a promising candidate for developing a highly sensitive, low-cost acetone sensor using capacitive-, QCM-, and resistive-based transduction. 

## Figures and Tables

**Table 1:** Comparison of thickness and mass of ZIF-8 films deposited on the sensors.

Device	ZIF-8 Film Thickness (µm)	ZIF-8 Mass (µg)
Cap-1	1.1	5.7
Cap-2	1	5.2
QCM-1	0.95	32
QCM-2	0.9	30

**Table 2:** Overview of the comparison of IDE- and QCM-based sensors.

Comparison Parameter	IDE Capacitor	QCM
Average sensitivity (per vol %)	1.3 (Δ*C*/*C*)	1.4 × 10^−5^ (Δ*f*/*f*)
Device size (mm^2^)	4.3	28.3
Detection limit (vol %)	0.0014	0.13
Avg. deviation (at 26.5 vol %)	4.4	2 × 10^−5^
Linearity	Nonlinear	Nonlinear
Energy (at 0.2 V_RMS_)	0.5 pJ	0.1 pJ
Hysteresis (vol %)	3	3
Selectivity (humidity)	Less	More
Temperature effect	More	Less
Response time at 20 sccm (min)	60	60
Cost ($)	1	10

**Table 3:** Comparison of the previously reported capacitive transduction-based acetone sensors.

Ref.	Sensing Material	Electrode Design	Sensitivity (Δ*C*/*C*)/vol %	Response Time (min)	Response Type	Hysteresis	Temperature (°C)
**This work**	**ZIF-8**	**IDE**	**30**	60 *	**Nonlinear**	**3 vol % in N_2_**	**RT**
[15] RSC Adv., 2017	poly(4-vinyl phenol)	IDE	9.7	2	Nonlinear	NR	RT
[16] Sensors 2015	HKUST-1	IDE	6.8 × 10^−4^	NR	Linear	NR	RT
[17] Sensors 2014	α-Fe_2_O_3_	IDE	340	0.32	Linear	NR	250
[18] Sens. Actuators A 2007	SWCNT	IDE	0.65	0.1	Nonlinear	NR	RT
[19] Science 2005	SWCNT + polycarbosilane	IDE	0.6	0.17	Nonlinear	NR	RT
[20] Meas. Sci. Technol. 1995	poly-*N*-(2-pyridy1)pyrro	Spiral	1.85	0.25	NR	NR	RT

Note: NR represents “not reported”; RT represents room temperature; * the response time reported is high due to the limitation of the maximum flow rate of the vapor delivery system (20 cm^3^/min).

**Table 4:** Comparison of the previously reported QCM transduction-based acetone sensors.

Ref.	Sensing Material	Sensitivity (Δ*f*/*f*)/vol %	Response Time (min)	Response Type	Hysteresis	Temperature (°C)
**This work**	**ZIF-8**	**2 × 10^−5^**	60 *	**Nonlinear**	**3 vol % in N_2_**	**RT**
[35] Sens. Actuators B 2016	Pd doped ZnO	3.8 × 10^−4^	0.2	Nonlinear	NR	RT
[36] Sens. Actuators B 2008	Imidazolium-based ionic liquids	1.6 × 10^−7^	1	Linear	NR	30
[37] Anal. Chim. Acta 2007	Thiophene	1.36 × 1^−5^	0.5	Linear	NR	RT
[38] Sens. Actuators B 2005	Calixarene	1 × 10^−5^	NR	NR	NR	RT
[39] Sens. Actuators B 2004	Ag+-ZSM-5 zeolite	9.3 × 10^−3^	4	Linear	NR	RT

Note: NR represents “not reported”; RT represents room temperature; * the response time reported is high due to the limitation of the maximum flow rate of the vapor delivery system (20 cm^3^/min).

**Table 5:** Comparison of the previously reported resistive transduction-based acetone sensors.

Ref.	Sensing Material	Sensitivity (Δ*R*/*R*)/vol %	Response Time (min)	Response Type	Temperature (°C)
**This work**	**ZIF-8**	**5.5 × 10^−2^**	60 *	**Nonlinear**	**RT**
[1] ACS Appl. Mater. Interfaces 2017	MXene	9.5	5	NR	RT
[2] Adv. Mater. 2016	ZIF-8 + ZnO	4 × 10^4^	6	Nonlinear	260
[3] IEEE Sens. J. 2016	Si-doped tungsten oxide	3.5 × 10^5^	4	Nonlinear	425
[4] RSC Adv. 2015	SnO_2_ reduced graphene oxide	43	2	Nonlinear	RT
[5] Mater. Lett. 2013	ZnO	2700	0.5	Nonlinear	310
[6] Anal. Chim. Acta 2012	Si doped WO_3_	15,000	1	Linear	350
[7] Sens. Actuators B 2011	SnO_2_	170	0.1	Nonlinear	200
[8] Sens. Actuators B 2011	Co doped ZnO	1500	0.14	Nonlinear	360
[9] Sens. Actuators B 2009	ZnO	3000	0.12	Nonlinear	320
[10] Sens. Actuators B 2008	ZnO	560	0.05	Nonlinear	300
[11] Sens. Actuators B 2006	nickel ferrite	0.3	3	Nonlinear	214
[12] Sens. Actuators B 2006	SnO_2_	25,000	0.34	Nonlinear	RT
[13] Sens. Actuators B 2000	CeO_2_	600	NR	NR	350

Note: NR represents “not reported”; RT represents room temperature; * the response time reported is high due to the limitation of the maximum flow rate of the vapor delivery system (20 cm^3^/min).
